# Partially hydrolyzed formula with high sn-2 palmitic acid on eosinophils and outcomes in preterm infants: PRIOR secondary analysis

**DOI:** 10.3389/fphar.2025.1724281

**Published:** 2026-01-12

**Authors:** Jialu Zhuang, Ye Liu, Qiongyu Liu, Jiaan Wang, Songlin Liu, Jing Wu, Yanqing Shen, Xuemin Wang, Hui Zhang, Li Yuan, Jinwen Chen, Ruizhen Geng, Zhiyan Zhan, Chuhan Dong, Fei Bei, Li Hong

**Affiliations:** 1 Department of Neonatology, Shanghai Children’s Medical Center, National Children’s Medical Center, Shanghai Jiaotong University School of Medicine, Shanghai, China; 2 Department of Neonatology, Women and Children’s Healthcare Hospital Of Linyi, Linyi, Shandong, China; 3 Department of Neonatology, Anhui Woman and Children’s Medical Center, Hefei, Anhui, China; 4 Department of Neonatology, Xuzhou Maternity and Child Health Care Hospital, Xuzhou, Jiangsu, China; 5 Department of Neonatology, Panyu Women and Children’s Medical Center, Guangdong Medical University (Guangzhou Panyu District Maternal and Child Health Hospital), Guangzhou, Guangdong, China; 6 Department of Clinical Nutrition, Shanghai Children’s Medical Center, National Children’s Medical Center, Shanghai Jiaotong University School of Medicine, Shanghai, China; 7 Clinical Research Center, Shanghai Children’s Medical Center, National Children’s Medical Center, Shanghai Jiaotong University School of Medicine, Shanghai, China; 8 Fujian Children’s Hospital (Fujian Branch of Shanghai Children’s Medical Center), College of Clinical Medicine for Obstetrics and Gynecology and Pediatrics, Fujian Medical University, Fuzhou, Fujian, China

**Keywords:** eosinophils, high sn-2 palmitic acid, immune modulation, nutritional intervention, partially hydrolyzed formula, preterm infants, randomized controlled trial

## Abstract

**Background:**

Preterm infants, with immature immune systems, are susceptible to type 2 inflammation, characterized by eosinophil recruitment, exacerbating intestinal and respiratory inflammation. Breastfeeding mitigates inflammation via sn-2 palmitic acid and immunomodulatory factors (e.g., IL-10), but formula feeding is often necessary due to clinical constraints. High sn-2 palmitic acid partially hydrolyzed formula (HPF) may reduce eosinophil-related inflammation by lowering protein immunogenicity, yet evidence is limited.

**Methods:**

This secondary analysis of the ongoing PRIOR parallel-group randomized controlled trial (ChiCTR2400093296) (evaluating formula safety and growth-related outcomes) included 90 preterm infants (gestational age <34 weeks or birth weight <2000 g), randomized to HPF (n = 45) or standard preterm formula (SPF) (n = 45) using computer-generated randomization with allocation concealment. Infants were enrolled from 1 July 2024, to 30 June 2025, and followed weekly during hospitalization and monthly after discharge until a corrected age of 3 months. Primary outcomes were hematological parameters (including eosinophil counts) at discharge; secondary outcomes included hospital stay duration, necrotizing enterocolitis (NEC) incidence, and anemia management. Generalized additive models assessed eosinophil levels relative to corrected gestational age and formula exposure duration.

**Results:**

Eighty infants completed the in-hospital phase (HPF, n = 41; SPF, n = 39). Groups were similar in gestational age, birth weight, sex, and Apgar score (all *p* > 0.05). HPF showed a steeper decline trend in eosinophil percentage after prolonged exposure (>25 days, *p* = 0.053), suggesting inflammation suppression. Hospital stay duration (HPF, 30 (17–38) days vs. SPF, 27 (20–36) days; *p* = 0.825) and NEC incidence (2.4% vs. 2.4%; *p* = 1.000) did not differ significantly.

**Conclusion:**

HPF demonstrates potential in reducing eosinophil-mediated inflammation in preterm infants but has no significant impact on hospital stay or NEC incidence. This secondary analysis supports optimizing preterm formulas and warrants further investigation into long-term immune benefits.

## 1 Introduction

Preterm infants (gestational age <34 weeks or birth weight <2000 g) are particularly susceptible to type 2 inflammation due to their immature immune systems. This Th2 cell-mediated response often promotes eosinophil recruitment and activation, thereby exacerbating intestinal and respiratory inflammation (Lao et al., 2022). Accumulating evidence indicates that a heightened type 2 inflammatory environment in preterm infants promotes eosinophil expansion, which contributing to abnormal lung development and early asthma-like features (Raftery et al., 2024). For instance, Lu et al., through the CHILD birth cohort, found that early-life food sensitization may precede inhalant sensitization, activating type 2 inflammatory pathways. Elevated eosinophil counts observed in some infants by age 1 indicate early inflammation onset, which is linked to long-term health risks (Lu et al., 2025).

Breastfeeding, widely recognized as the “gold standard” for infant nutrition, helps mitigate type 2 inflammation via its unique lipid profile, in which approximately 70% of palmitic acid is esterified at the sn-2 position of triacylglycerols (Shoji et al., 2024), along with immunomodulatory factors (e.g., IL-10), which collectively promote gut barrier integrity and immune tolerance (Ballard and Morrow, 2013). However, maternal lactation difficulties or preterm birth often necessitate formula feeding (Odom et al., 2013). Standard preterm formulas (SPF) typically contain intact proteins, which are potentially increasing feeding intolerance and inflammation risks. In contrast, partially hydrolyzed formulas (pHF) employ enzymatic hydrolysis to partially break down proteins, thereby enhancing digestibility and reducing immunogenicity (Vandenplas et al., 2019). Although some studies suggest that pHF may prevent atopic diseases (Greer et al., 2019), the evidence remains inconclusive, and pHF is not recommended for allergy treatment (Greer et al., 2019). Moreover, formulas enriched with sn-2 palmitic acid, which mimic breast milk’s lipid structure, may reduce eosinophil-related inflammation. However, randomized controlled trials specifically evaluating eosinophil levels in relation to corrected gestational age and formula exposure duration in preterm infants are scarce (Lo et al., 2025).

The PRIOR study, a multicenter, open-label, parallel-group randomized controlled trial, compares a high sn-2 palmitic acid partially hydrolyzed formula (HPF) with standard preterm formula (SPF) regarding their effects on hematological inflammatory markers (notably eosinophils) and clinical outcomes in preterm infants. This study aims to elucidate HPF’s potential to suppress eosinophil-mediated inflammation, shorten hospitalization, and optimize anemia management, providing evidence for tailored nutritional interventions to reduce long-term immune-related complications.

## 2 Materials and methods

### 2.1 Study design and participants

#### 2.1.1 Study design

This multicenter, open-label, parallel-group randomized controlled trial—the PRIOR study (Preterm Randomized Intervention with OPO Structured-Lipid)—was conducted at five medical institutions in China: Shanghai Children’s Medical Center, Women and Children’s Health Care Hospital Of Linyi, Anhui Woman and Children’s Medical Center, Xuzhou Maternity and Child Health Care Hospital, and Panyu Maternal and Panyu Women and Children’s Medical Center. We enrolled infants (gestational age <34 weeks or birth weight <2000 g) born between 1 July 2024, and 30 June 2025, who required formula feeding within the first 7 days of life. This secondary analysis is limited to in-hospital outcomes and the full study follow-up is scheduled to end on 31 December 2025. Eligible infants were randomly assigned to one of two groups: the experimental group received a partially hydrolyzed formula in which 60% of the palmitic acid was esterified at the sn-2 position, while the control group received a standard vegetable oil-based preterm formula. The two formulas were comparable in macronutrient composition.

All participants were followed until meeting discharge criteria (Phase 1, completed). Following this, a further follow-up extending to a corrected age of 3 months is being conducted based on parental willingness and is currently underway.

The trial was conducted in accordance with the Declaration of Helsinki and was approved by the Ethics Committee of Shanghai Children’s Medical Center and all collaborating centers (SCMCIRB-K2024124-1). Written informed consent was obtained from the legal guardian of each infant prior to enrollment. This trial is registered in the Chinese Clinical Trial Registry (Registration number: ChiCTR2400093296).

#### 2.1.2 Participants

This study enrolled preterm infants with gestational age <34 weeks or birth weight <2000 g, exclusively formula-fed based on maternal decision. Inclusion required postnatal age 1–7 days, anticipated enteral feeding within the first week, and breast milk intake ≤20% of total feeding. Guardians provided written informed consent.

Exclusion criteria comprised: severe congenital anomalies or chromosomal abnormalities; prior or planned surgical intervention; severe intracranial hemorrhage (grade III+) or hydrocephalus; imminent mortality risk within 72 h; participation in other interventional trials; or parental refusal.

Withdrawal criteria included: death or discharge before full enteral nutrition; protocol non-compliance; guardian request; or investigator-determined medical necessity.

#### 2.1.3 Randomization and blinding

The randomization list was generated by an independent statistician using SAS software to produce a computer-generated random number table, with competitive recruitment across study centers. For multiple pregnancies (e.g., twins), randomization was performed by twin pair as a cluster unit to ensure that both infants from the same pregnancy were assigned to the same intervention group, thereby preventing cross-contamination in feeding. Each randomization unit (singleton or twin pair) was allocated in a 1:1 ratio to the HPF or SPF group. The statistician was not involved in patient recruitment, clinical care, or data analysis.

Allocation concealment was achieved using sequentially numbered, sealed envelopes. Research nurses opened the corresponding envelope in sequential order based on the randomization number to determine group assignment only after obtaining written informed consent and confirming eligibility. Due to observable differences in formula appearance and packaging, the trial was open-label. However, laboratory analysts assessing hematological outcomes were blinded to group allocation.

#### 2.1.4 Data collection and definitions

All baseline and clinical data were prospectively documented using a standardized case report form (CRF), with parallel entry into an electronic data capture (EDC) system. Key infant characteristics, including gestational age (determined in completed weeks and days) and birth weight (measured to the nearest gram), were documented with particular precision. Maternal antenatal morbidities—including circulatory disorders (e.g., hypertensive diseases of pregnancy), endocrine disorders (e.g., gestational diabetes, thyroid dysfunction), late-pregnancy infections, immune disorders, cervical-placental conditions, and other significant comorbidities—were classified based on standard diagnostic criteria and physician documentation in the medical record.

#### 2.1.5 Feeding guidelines

To ensure consistency in feeding practices across multiple centers and to mitigate potential outcome bias attributable to inter-center feeding variability, a standardized feeding protocol was developed in accordance with current evidence-based guidelines for preterm infant nutrition (22,23). Parenteral nutrition (PN) was initiated within the first 48 h of life when clinically feasible (i.e., hemodynamic stability and established central or peripheral venous access). Enteral feeding was commenced as soon as clinical stability was achieved, typically within 24–72 h postnatally. The target total enteral intake (via gavage or oral route) was 150 mL/kg/day and 120 kcal/kg/d, with daily increments of 20–30 mL/kg/day. Final adjustments to the feeding regimen were individualized by the attending physician or higher-level clinical supervisor based on feeding tolerance (e.g., gastric residuals, abdominal distension, vomiting), gastrointestinal signs, and established preterm feeding guidelines.

### 2.2 Outcomes

This secondary analysis of the completed in-hospital phase of the ongoing PRIOR study evaluated a comprehensive set of hematological and clinical outcomes to elucidate the early-life effects of nutritional intervention, with particular emphasis on eosinophil levels as potential markers of inflammation and immune modulation.

#### 2.2.1 Eosinophil levels in relation to corrected gestational age and formula exposure

The primary focus was on exploring eosinophil (EOS) absolute counts (EOS_num, ×10^9^/L) and percentages (EOS_pct, %) to explore their dynamics, which may reflect allergic or inflammatory processes potentially influenced by preterm maturation and formula feeding. These analyses specifically examined the relationship of EOS levels with corrected gestational age (CGA) and the formula exposure duration (days). CGA was categorized into grades as follows: Grade 1 (<30 weeks), Grade 2 (30–31 weeks), Grade 3 (32–33 weeks), Grade 4 (34–35 weeks), Grade 5 (36–37 weeks), and Grade 6 (>37 weeks).

#### 2.2.2 Other outcomes

Additional primary outcomes focused on hematological profiles at discharge, including white blood cell count, hemoglobin level, platelet count, and differential counts of neutrophils, lymphocytes, and eosinophils. Venous blood samples for complete blood count were collected at the bedside by trained laboratory personnel after obtaining family consent, to avoid potential hemoconcentration bias associated with capillary sampling.

Secondary outcomes encompassed key clinical endpoints: duration of hospitalization (days), incidence of necrotizing enterocolitis (diagnosed according to Bell’s staging criteria), and anemia prevalence and management (categorized as mild untreated, transfusion-requiring, or managed with iron supplementation only), assessed from enrollment to discharge.

### 2.3 Statistical analysis

All statistical analyses were performed using R software (version 4.2.1). Continuous variables were summarized as means ± standard deviations, and categorical variables as frequencies and percentages. Baseline demographic and clinical characteristics were compared between the HPF and SPF groups using independent t-tests for continuous data and chi-square tests or Fisher’s exact tests for categorical data, with a significance level set at p < 0.05. Secondary clinical outcomes, such as duration of hospitalization and incidence of necrotizing enterocolitis (NEC, Bell’s stage ≥ II), were compared using t-tests and Fisher’s exact tests, respectively. Anemia prevalence and management categories (mild untreated, transfusion-requiring, or iron supplementation only) were evaluated with chi-square tests.

Generalized additive models (GAMs) were employed using the mgcv package in R (version 4.2.1) to capture non-linear trends in eosinophil absolute count (EOS_num, ×10^9^/L) and percentage (EOS_pct, %). Model fit was assessed using deviance explained and visualized with ggplot2, incorporating 50% standard error confidence intervals to reflect variability.

The primary analyses were conducted using a per-protocol approach, including only infants who completed the in-hospital phase without major protocol violations (n = 80). An intention-to-treat analysis was not performed due to the secondary nature of this in-hospital analysis and the limited number of withdrawals with incomplete outcome data.

## 3 Results

### 3.1 Study cohort

A total of 90 infants were enrolled and randomized using a computer-generated sequence created by a statistician with SAS software. Participants were allocated in a 1:1 ratio to either the high-OPO, partially hydrolyzed preterm formula (HPF) group (n = 45) or the standard preterm formula (SPF) group (n = 45). Randomization was performed in a competitive manner across the participating centers.

Following randomization, one infant in the HPF group was withdrawn after the legal guardian withdrew informed consent before the initiation of formula feeding. Thus, 89 infants (44 in the HPF group and 45 in the SPF group) were fed the assigned feeding regimen.

**FIGURE 1:**
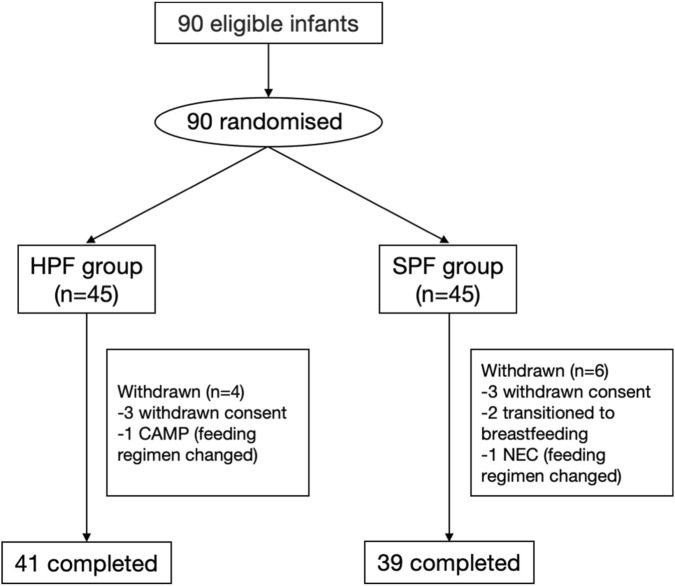
Flow diagram of the PRIOR study. HPF, high-OPO partially hydrolyzed preterm formula; SPF, standard preterm formula; CAMP, cow’s milk protein allery; NEC, necrotizing enterocolitis.

During the in-hospital Phase 1, a total of 9 infants discontinued the study. In the HPF group, three infants withdrew: two due to withdrawn consent and one due to feeding intolerance that required a switch to an extensively hydrolyzed formula. In the SPF group, six infants withdrew: three due to withdrawn consent, two due to parental decision to transition to exclusive breastfeeding, and one who developed necrotizing enterocolitis (NEC) and required a change in feeding regimen. Consequently, 80 infants (41 HPF, 39 SPF) completed the in-hospital phase (Figure 1).

### 3.2 Baseline characteristics

Table 1 summarizes the baseline demographic and clinical characteristics of the randomised infants. After excluding six subjects who withdrew informed consent in the absence of clear medical reasons, baseline analysis was performed on the remaining infants. The two groups were comparable in most baseline characteristics. Specifically, no significant differences were observed in gestational age (HPF: 32 ± 2.3 weeks vs. SPF: 32.1 ± 1.8 weeks; *p* = 0.804), birth weight (HPF: 1,614 ± 295 g vs. SPF: 1,643 ± 318 g; *p* = 0.188), or sex distribution (*p* = 0.138). Notably, 11 infants had birth weight ≤1,250 g (HPF: n = 5, 1,080–1,250 g; SPF: n = 6, 950–1,250 g), with the lowest being 950 g (GA 29+2 weeks) in the SPF group and 1,080 g (GA 29+0 weeks) in the HPF group. However, significant differences were noted in delivery mode and birth type: the SPF group had a higher rate of cesarean section (95.3% vs. 76.2%, *p* = 0.026) and multiple births (59.5% vs. 38.1%, *p* = 0.049). The baseline maternal conditions, including hypertensive disorders of pregnancy and other antenatal morbidities, were comparable between the two groups, with no statistically significant differences.

**TABLE 1:** Baseline characteristics at randomization.

Characteristic	HFP group (n = 42)	SFP group (n = 42)	*P* value
Sex, male	22 (52.4%)	16 (38.1%)	0.188^a^
Gestational age at birth (weeks)	32 ± 2.3	32.1 ± 1.8	0.804^b^
Birth weight (g)	1,614 ± 295	1,643 ± 318	0.660^b^
Cesarean section	32 (76.2%)	40 (95.2%)	0.026^c^ ^,^ ^d^
Multiple birth	16 (38.1%)	25 (59.5%)	0.049^a^ ^,^ ^d^
5 min Apgar score	8.5 (8–9)	9 (8–10)	0.317^e^
Maternal antenatal morbidity, n (%)			
None	15 (35.7%)	9 (21.4%)	0.347^a^
Isolated system morbidity	16 (38.1%)	19 (45.2%)	—
Complex multi-system morbidity	11 (26.2%)	14 (33.3%)	—
Specific maternal antenatal comorbidities			
Hypertensive disorders of pregnancy	9 (21.4%)	16 (38.1%)	0.095^a^
Gestational Endocrinopathies	16 (38.1%)	20 (47.6%)	0.378^a^
Infectious morbidity	3 (7.1%)	4 (9.5%)	1.000^c^
Autoimmune/Rheumatic diseases	3 (7.1%)	3 (7.1%)	1.000^c^
Cervical-placental disorders	4 (9.5%)	2 (4.8%)	0.676^c^
Other comorbid conditions	4 (9.5%)	4 (9.5%)	1.000^c^

Data are mean ± SD, for normally distributed continuous variables or median (IQR) for non-normally distributed continuous variables, and n (%) for categorical variables. HPF, high-OPO, partially hydrolyzed preterm formula; SPF, standard preterm formula.

^a^ χ2 test.

^b^ Student’s t-test.

^c^ Fisher’s exact test.

^d^ *p* < 0.05, considered statistically significant.

^e^ Wilcoxon rank-sum test.

### 3.3 Eosinophil levels in relation to corrected gestational age and formula exposure

#### 3.3.1 Influence of corrected gestational age on eosinophil levels

**FIGURE 2:**
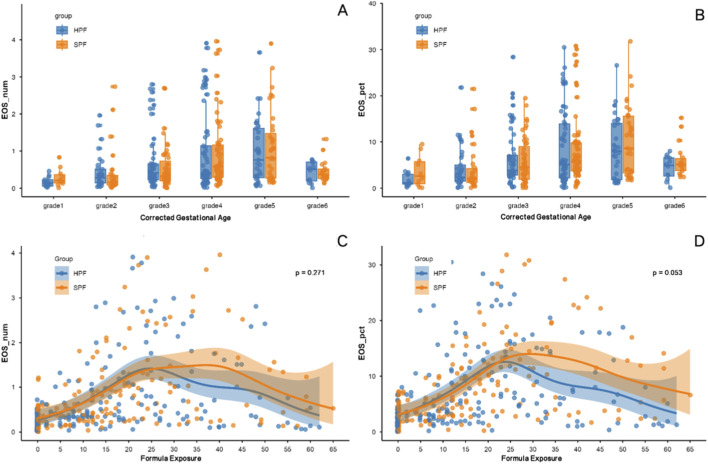
Eosinophil Levels in Preterm Infants Across Corrected Gestational Age and Formula Exposure Duration. **(A)** Eosinophil Absolute Count (EOS_num) by Corrected Gestational Age: Scatter plots show eosinophil absolute count (EOS_num, ×10^9^/L) across corrected gestational age (CGA) grades (Grade 1: <30 weeks; Grade 2: 30–31 weeks; Grade 3: 32–33 weeks; Grade 4: 34–35 weeks; Grade 5: 36–37 weeks; Grade 6: >37 weeks) for HPF (blue) and SPF (orange) groups. **(B)** Eosinophil Percentage (EOS_pct) by Corrected Gestational Age: Scatter plots display eosinophil percentage (EOS_pct, %) across CGA grades for HPF and SPF. **(C)** EOS_num Trends by Formula Exposure Duration: Fitted curves depict eosinophil absolute count (EOS_num, ×10^9^/L) trends over formula exposure duration (days) for HPF (blue) and SPF (orange), with 50% confidence intervals. **(D)** EOS_pct Trends by Formula Exposure Duration: Fitted curves illustrate eosinophil percentage (EOS_pct, %) trends over formula exposure duration, with 50% confidence intervals.

As illustrated in Figure 2, EOS_num and EOS_pct exhibited distinct patterns across CGA grades in the HPF and SPF groups. For EOS_num (Figure 2A), values were generally low in early grades (Lao et al., 2022; Raftery et al., 2024), with a progressive increase peaking around grades 3–4 (approximately 32–35 weeks CGA), followed by a decline in grades 5–6. This pattern was more pronounced in the SPF group, where higher variability and occasional outliers were observed in mid-grades, potentially indicating greater sensitivity to maturational changes or formula components. Similarly, EOS_pct (Figure 2B) showed a comparable trend, with percentages rising to peaks in grades 3–5 before decreasing, again with notable elevations in the SPF group during these periods. These observations suggest that eosinophil levels may be influenced by gestational maturation, with mid-preterm stages associated with heightened EOS activity, possibly mitigated to a greater extent by HPF compared to SPF.

#### 3.3.2 Impact of formula exposure duration on eosinophil trends

Generalized additive models (GAMs) were used to evaluate non-linear trends in eosinophil absolute counts (EOS_num) and percentages (EOS_pct) as a function of formula exposure duration (Figure 2, bottom panels).

For EOS_num, both HPF and SPF exhibited similar trajectories up to 25 days, with an initial rise peaking around 20–25 days, followed by a decline. Beyond 25 days, HPF showed a more substantial reduction compared to SPF (*p* = 0.271), indicating a potential advantage of HPF in suppressing eosinophil counts over prolonged exposure.

For EOS_pct, trends were comparable between groups before 25 days, with both peaking around 20–25 days (e.g., HPF 30.5%, SPF 34%). After 25 days, HPF demonstrated a steeper decline (*p* = 0.053), suggesting enhanced modulation and a possible reduction in non-IgE-mediated inflammation compared to SPF.

These patterns suggest subtle, time-dependent effects of formula exposure on eosinophil dynamics, particularly in percentages, with HPF showing a trend toward lower levels that aligns with its potential to better manage inflammation.

### 3.4 Other hematological outcomes

Hematological parameters serve as sensitive indicators of nutritional status, inflammatory responses, and developmental progress in preterm infants. As summarized in Table 2, all baseline hematological values were comparable between the HPF and SPF groups.

**TABLE 2:** Hematological parameters at enrollment and discharge.

Parameter	HFP group	N	SFP group	N	*P*-value
Eosinophil count (×10^9^/L)					
Enrollment	0.18 (0.12–0.33)	42	0.26 (0.15–0.42)	42	0.140^c^
Discharge	0.46 (0.28–0.83)	40	0.6 (0.31–1.03)	38	0.296^c^
White blood cell count (×10^9^/L)					
Enrollment	8.57 (6.74–10.48)	42	8.77 (7.43–10.93)	42	0.534^c^
Discharge	9.65 ± 2.93	40	8.89 (7.56–10.65)	38	0.444^c^
Hemoglobin (g/L)					
Enrollment	159.5 ± 22.4	42	161.8 ± 31.3	42	0.704^b^
Discharge	113.3 ± 22.0	40	108 .0 ± 21.1	38	0.282^b^
Platelet count (×10^9^/L)					
Enrollment	253 (201–323)	42	241 ± 75	42	0.460^c^
Discharge	351 (302–432)	40	368 (327–460)	38	0.217^c^
Neutrophil count (×10^9^/L)					
Enrollment	4.2 (3.02–5.6)	42	3.95 (2.92–6.72)	42	0.900^c^
Discharge	3 (1.82–4.28)	40	2.38 (1.69–3.11)	38	0.192^c^
Lymphocyte count (×10^9^/L)					
Enrollment	3.13 ± 1.25	42	3.13 (2.38–4.69)	42	0.204^c^
Discharge	4.76 (3.86–5.85)	40	4.7 ± 1.23	38	0.715^c^
Monocyte count (×10^9^/L)					
Enrollment	0.94 (0.66–1.31)	42	0.84 (0.59–1.34)	42	0.436^c^
Discharge	1.13 (0.7–1.35)	40	0.86 (0.66–1.06)	38	0.105^c^

Data are mean ± SD, for normally distributed continuous variables or median (IQR) for non-normally distributed continuous variables. HPF, high-OPO, partially hydrolyzed preterm formula; SPF, standard preterm formula.

^a^ values.

^b^ Student’s t-test.

^c^ Wilcoxon rank-sum test. Normality assessed by Shapiro-Wilk test.*p < 0.05, considered statistically significant.

Analysis using repeated-measures ANOVA showed no significant interaction between feeding group and time for any hematological parameter (all p > 0.05), except for trends observed in eosinophil levels as detailed above, indicating that changes from enrollment to discharge did not differ between the two feeding regimens. The interaction statistics were as follows: white blood cell count (F (1,76) = 0.56, *p* = 0.456), hemoglobin (F (1,76) = 1.75, *p* = 0.190), platelet count (F (1,76) = 1.27, *p* = 0.263), neutrophil count (F (1,76) = 0.75, *p* = 0.390), lymphocyte count (F (1,76) = 1.26, *p* = 0.265), monocyte count (F (1,76) = 1.06, *p* = 0.307), and eosinophil count (F (1,76) = 0.04, *p* = 0.850).

The characteristic postnatal decrease in hemoglobin and increase in platelet counts occurred to a similar extent in both groups, which is consistent with normal maturation and further underscores the similarity in outcomes between the groups.

### 3.5 Clinical outcomes

Clinical secondary outcomes are presented in Table 3. The duration of hospitalization was comparable between the HPF and SPF groups (30 (17–38) days vs. 27 (20–36) days; *p* = 0.825). The incidence of necrotizing enterocolitis (Bell’s stage ≥ II) was low and did not differ between groups (2.4% vs. 2.4%; *p* = 1.000).

**TABLE 3:** Secondary clinical outcomes in the in-hospital phase (phase 1).

Parameter	HFP group (n = 42)	SFP group (n = 42)	*P* value
Length of hospital stay (days)	30 (17–38)	27 (20–36)	0.825^c^
Necrotizing enterocolitis	1 (2.4%)	1 (2.4%)	1.000^a^
Anemia, overall	14 (33.3%)	10 (23.8%)	0.334^a^
-Transfusion-requiring	2 (4.8%)	2 (4.8%)	1.000^a^
-Iron supplementation	1 (2.4%)	4 (9.5%)	0.360^a^
-Mild, untreated^b^	11 (26.2%)	4 (9.5%)	0.085^a^

Data are mean ± SD, for normally distributed continuous variables or median (IQR) for non-normally distributed continuous variables, and n (%) for categorical variables.

^a^ Fisher’s exact test. Normality assessed by Shapiro-Wilk test.

^b^ Mild, untreated anemia was defined as anemia not requiring transfusion or oral iron supplementation. *p < 0.05, considered statistically significant.

^c^ Wilcoxon rank-sum test.

The overall incidence of anemia and its distribution across management strategies—including mild untreated, transfusion-requiring, and iron supplementation-only—showed no statistically significant differences between groups (all *p* > 0.05).

## 4 Discussion

This study is a secondary analysis of the PRIOR randomized controlled trial, which evaluated a high sn-2 palmitic acid partially hydrolyzed formula (HPF) versus standard preterm formula (SPF) on inflammatory markers and clinical outcomes in preterm infants (gestational age <34 weeks or birth weight <2,000 g). In five Chinese medical centers, we randomly assigned 90 infants to HPF or SPF groups and focused on in-hospital hematological parameters (especially eosinophil levels), length of stay, necrotizing enterocolitis (NEC) incidence, and anemia management. We used a multicenter, open-label design with standardized data collection and blinded statistical analysis to ensure reliability.

Results showed a steeper decline in eosinophil percentage (EOS_pct) in the HPF group after more than 25 days of exposure (p = 0.053), suggesting possible inflammation suppression through reduced protein immunogenicity or breast milk-like lipid structure. Generalized additive models (GAM) revealed lower EOS peaks in HPF at corrected gestational age 32–35 weeks, indicating time-dependent anti-inflammatory effects. However, no differences were found in length of stay (30 (17–38) vs. 27 (20–36) days, *p* = 0.825) or NEC incidence (2.4% vs. 2.4%, *p* = 1.000). These preliminary findings suggest HPF’s potential in modulating eosinophil-mediated inflammation, offering insights for optimizing preterm formulas.

Both HPF and SPF groups showed an initial rise and subsequent fall in eosinophil counts (EOS_num) and percentages (EOS_pct) with increasing corrected gestational age (CGA), peaking at 32–35 weeks (grades 3–4) before declining. Although no statistical group differences emerged, HPF had a faster EOS_pct decline after 25 days of exposure, suggesting potential advantages in suppressing inflammation in preterm infants. This pattern reflects preterm immune development, where Th2-biased responses make infants prone to eosinophil-mediated type 2 inflammation during rapid growth phases (32–35 weeks), possibly due to immature gut barriers or formula stimulation (Lao et al., 2022; Raftery et al., 2024). Ballabh et al. found eosinophil activation linked to bronchopulmonary dysplasia, implying elevated levels raise wheezing risk and highlight the need for early control (Broström et al., 2007). SPF showed greater peak variability, suggesting standard formulas may trigger activation more easily, while HPF’s hydrolyzed proteins mitigate this by lowering immunogenicity (Vandenplas et al., 2019).

We further investigated potential confounding factors that might influence eosinophil levels, particularly antibiotic usage, which is known to modulate immune responses. Supplementary analysis revealed no significant differences in eosinophil counts (EOS1 and EOS2) between infants who received antibiotics and those who did not, within either the HPF or SPF groups (all p > 0.05; Supplementary Table S3). This suggests that the observed eosinophil dynamics were primarily driven by developmental maturation and formula intervention rather than antibiotic exposure.

HPF’s anti-inflammatory effects may stem from synergy between high sn-2 palmitic acid and whey hydrolysates. Yaron et al. reported improved gut microbiota and reduced inflammatory markers with such formulas (Yaron et al., 2013). Whey hydrolysates may further suppress eosinophils by modulating microbiota; Li et al.'s *in vitro* model showed increased short-chain fatty acids (SCFA) and beneficial bacteria (e.g., *Bacteroides*), lowering Firmicutes/Bacteroidetes ratio for an anti-inflammatory environment (Feng et al., 2022).

Despite no overt allergies observed, subclinical eosinophil rises matter, as they may signal long-term risks like asthma. Johannsen et al. reviewed infant eosinophil elevation linked to allergy risk (HR ≈ 1.3–1.5), stressing early microbiota interventions (West, 2014). West et al. noted dysbiosis exacerbates Th2 pathways, while SCFA can ease it (Musso et al., 2020). HPF’s time-dependent benefits suggest prolonged use optimizes microbiota and reduces risks, but non-significant differences may limit samples or variability—larger cohorts and follow-up are needed.

Regarding other nutritional outcomes, the overall incidence of anemia and its management strategies were comparable between the HPF and SPF groups. This suggests that, within the scope of this study, the modified formula did not adversely affect iron status or anemia management. The primary focus thus remains on its potential immunomodulatory effects.

The preliminary observations provide a reference for preterm nutrition interventions, though clinical applicability needs further validation. HPF’s inflammation modulation positions it as a potential breast milk substitute for immune management in resource-limited settings. This is consistent with Stróżyk et al. (2020)’s systematic review, which—based on RCTs—shows hydrolyzed formulas enhance tolerance in cow’s milk protein allergy infants but requires individual evaluation (Stróżyk et al., 2020). In China’s multicenter setting, the results could inform early guideline adjustments, such as piloting HPF to track feeding compliance. Still, widespread use demands stronger evidence to prevent nutritional risks, like anemia imbalances.

GAM analysis of exposure patterns underscores nonlinear approaches’ role in nutritional immunology, building on understanding of early feeding’s immune impacts (Feng et al., 2022). It resonates with Greer et al. (2019) guidelines, which prudently endorse hydrolyzed formulas for high-risk infants yet urge more RCTs for preterms (Greer et al., 2008). The anemia findings also signal the need to address nutrient interaction complexity in formula design.

This study has limitations. The study’s statistical power was limited by the small sample size (n = 80 completers) and a per-protocol analysis, and further constrained by post-randomization dropouts that could introduce bias, particularly for assessing rare outcomes like NEC. Second, the open-label design risks bias, as group knowledge could affect feeding or decisions. Third, analysis stopped at discharge, with long-term effects still under follow-up. Fourth, preterm sampling challenges prevented molecular markers (e.g., IL-10), limiting mechanistic insights from blood counts alone. Fifth, HPF integrates high sn-2 palmitic acid and partial protein hydrolysis, precluding isolation of their individual effects; despite extensive efforts, we were unable to procure in-hospital preterm formulas with either modification in isolation during the study. Finally, while we accounted for antibiotic usage, other unmeasured confounders such as viral infections or detailed maternal factors could potentially influence eosinophil levels. Results from Chinese hospitals may not generalize elsewhere. Despite these limitations, our study provide a foundation for preliminary evidence, still needing larger trials for validation and expansion.

It is important to note that the observed trend in eosinophil reduction, while suggestive, did not reach conventional statistical significance (e.g., p = 0.053 for EOS_pct decline). This lack of definitive statistical evidence may be attributed to the limited sample size and statistical power of our study, as previously noted in the limitations. Consequently, while the biological plausibility and the temporal pattern revealed by GAM are encouraging, they must be interpreted as preliminary. Future studies with larger cohorts are required to confirm whether this trend translates into a statistically significant and clinically meaningful effect.

## 5 Conclusion

This analysis indicated a potential trend towards a faster decrease in eosinophil levels with prolonged use of the high sn-2 palmitate, partially hydrolyzed formula (HPF) show, although this did not reach statistical significance in our limited sample. No association was found between eosinophil levels and antibiotic use. However, these changes did not lead to shorter hospital stays or lower NEC rates. HPF could be a useful option to support infant nutrition, but more studies are needed to confirm its benefits.

## Data Availability

The raw data supporting the conclusions of this article will be made available by the authors, without undue reservation.
