# Supplementary material for: Partially hydrolyzed formula with high sn-2 palmitic acid on eosinophils and outcomes in preterm infants: PRIOR secondary analysis
Source: Front Pharmacol. 2026 Jan 12;16:1724281. doi: 10.3389/fphar.2025.1724281 (PMC12832879; doi:10.3389/fphar.2025.1724281)
Supplement: Supplementary file 1 [file Table1.docx]

**Supplementary Table S1. Eosinophil Counts by Study Center**

|  |  | **Intervention Group** | |
| --- | --- | --- | --- |
| **Center** | **Time Point** | **HPF** | **SPF** |
| Center 1 | Eosinophils Enrollment (×10⁹/L) | 0.29 ± 0.21 (n=10) | 0.3 (0.13–0.53) (n=17) |
| Center 1 | Eosinophils Discharge (×10⁹/L) | 0.5 (0.26–0.69) (n=10) | 0.7 ± 0.43 (n=14) |
| Center 2 | Eosinophils Enrollment (×10⁹/L) | 0.17 ± 0.14 (n=10) | 0.18 ± 0.12 (n=10) |
| Center 2 | Eosinophils Discharge (×10⁹/L) | 0.45 ± 0.28 (n=9) | 0.42 ± 0.16 (n=10) |
| Center 3 | Eosinophils Enrollment (×10⁹/L) | 0.26 ± 0.18 (n=17) | 0.17 (0.08–0.2) (n=5) |
| Center 3 | Eosinophils Discharge (×10⁹/L) | 0.42 (0.28–0.94) (n=15) | 1.08 (0.94–1.16) (n=3) |
| Center 4 | Eosinophils Enrollment (×10⁹/L) | 0.24 (0.19–0.26) (n=6) | 0.5 (0.32–1) (n=6) |
| Center 4 | Eosinophils Discharge (×10⁹/L) | 0.7 (0.47–0.98) (n=5) | 0.9 (0.67–0.99) (n=4) |
| Center 5 | Eosinophils Enrollment (×10⁹/L) | 0.18 (0.18–0.18) (n=1) | 0.32 ± 0.17 (n=7) |
| Center 5 | Eosinophils Discharge (×10⁹/L) | 0.34 (0.34–0.34) (n=1) | 0.93 ± 0.8 (n=7) |
| Data are mean ± SD for normally distributed continuous variables or median (IQR) for non-normally distributed continuous variables. | | | |
