# Supplementary material for: Partially hydrolyzed formula with high sn-2 palmitic acid on eosinophils and outcomes in preterm infants: PRIOR secondary analysis
Source: Front Pharmacol. 2026 Jan 12;16:1724281. doi: 10.3389/fphar.2025.1724281 (PMC12832879; doi:10.3389/fphar.2025.1724281)
Supplement: Supplementary file 2 [file Table2.docx]

**Supplementary Table S2. Nutritional Composition Comparison of the Two Experimental Infant Formulas**

| Nutritional Per 100g | **HPF** | **SPF** |
| --- | --- | --- |
| Engrgy/(kJ) | 2110 | 2084 |
| Protein/(g) | 14.56 | 14.4 |
| Fat/(g) | 25.63 | 25.9 |
| Linoleic Acid/(g) | 3.53 | 3.878 |
| α-Linoleic Acid/(mg) | 393 | 438 |
| Carbohydrate/(g) | 53.8 | 51.825 |
| Sodium/(mg) | 283 | 269 |
| Potassium/(mg) | 496 | 605 |
| Copper/(μg) | 553.7 | 770 |
| Magnesium/(mg) | 47.2 | 50 |
| Iron/(mg) | 10.25 | 9.4 |
| Zinc/(mg) | 6.15 | 5.6 |
| Manganese/(μg ) | 70.7 | 50 |
| Calcium/(mg) | 656 | 760 |
| Phosphorus/(mg) | 381.4 | 445 |
| Iodine/(μg) | 143.5 | 136 |
| Chloride/(mg) | 439 | 430 |
| 1,3-dioleoyl-2-palmitoylglycerol/(g) | 4.2 | / |
