# Supplementary material for: Partially hydrolyzed formula with high sn-2 palmitic acid on eosinophils and outcomes in preterm infants: PRIOR secondary analysis
Source: Front Pharmacol. 2026 Jan 12;16:1724281. doi: 10.3389/fphar.2025.1724281 (PMC12832879; doi:10.3389/fphar.2025.1724281)
Supplement: Supplementary file 3 [file Table3.docx]

|  | **Intervention Groups** | | | |  | |
| --- | --- | --- | --- | --- | --- | --- |
|  | **HPF Group** | | **SPF Group** | |  | |
| **Time Point** | **No Antibiotics** | **With Antibiotics** | **No Antibiotics** | **With Antibiotics** | **P-value1** | **P-value2** |
| Eosinophils 1 (×10⁹/L) | 0.26 ± 0.15 (n=19) | 0.17 (0.07–0.3) (n=23) | 0.29 (0.23–0.5) (n=14) | 0.21 (0.13–0.34) (n=28) | 0.28 | 0.17 |
| Eosinophils 2 (×10⁹/L) | 0.48 (0.34–0.94) (n=17) | 0.38 (0.22–0.7) (n=23) | 0.81 (0.34–1.03) (n=13) | 0.53 (0.3–1.01) (n=25) | 0.12 | 0.6 |
| Data are mean ± SD for normally distributed continuous variables or median (IQR) for non-normally distributed continuous variables.   P-values from Wilcoxon rank-sum test comparing eosinophil levels between antibiotic usage groups within each intervention group.  EOS1: Enrollment eosinophil count; EOS2: Discharge eosinophil count. | | | | | | |
